# Echocardiography Abnormal Findings and Laboratory Operations during the COVID-19 Pandemic at a High Volume Center in New York City

**DOI:** 10.3390/healthcare8040534

**Published:** 2020-12-03

**Authors:** Li Pang, Eric P. Stahl, Kana Fujikura, Michelle Chen, Weijia Li, Ming Zhang, Jeffrey M. Levsky, Mark I. Travin, Edwin C. Ho, Ythan Goldberg, Cynthia C. Taub

**Affiliations:** 1 Department of Medicine, Jacobi Medical Center, Bronx, NY 10461, USA; lpang1@montefiore.org (L.P.); wli15@montefiore.org (W.L.); 2 Department of Medicine, Division of Cardiology, Montefiore Medical Center, Albert Einstein College of Medicine, Bronx, NY 10467, USA; estahl@montefiore.org (E.P.S.); kdagost@montefiore.org (K.F.); mtravin@montefiore.org (M.I.T.); eho1@montefiore.org (E.C.H.); ygoldber@montefiore.org (Y.G.); 3 Department of Health and Human Services, National Heart, Lung and Blood Institute, National Institutes of Health, Bethesda, MD 20892, USA; 4 Department of Medicine, Montefiore Medical Center, Albert Einstein College of Medicine, Bronx, NY 10467, USA; mchen4@montefiore.org (M.C.); mizhang@montefiore.org (M.Z.); 5 Department of Radiology, Montefiore Medical Center, Albert Einstein College of Medicine, Bronx, NY 10467, USA; jlevsky@montefiore.org

**Keywords:** echocardiography, COVID-19, healthcare

## Abstract

(1) Background: This study sought to explore how the novel coronavirus (COVID-19) pandemic affected the echocardiography (TTE) laboratory operations at a high volume medical center in New York City. Changes in cardiac imaging study volume, turn-around time, and abnormal findings were analyzed and compared to a pre-pandemic period. (2) Methods: Volume of all cardiac imaging studies and TTE reports between 11 March 2020 to 5 May 2020 and the same calendar period in 2019 were retrospectively identified and compared. (3) Results: During the pandemic, our center experienced a 46.72% reduction in TTEs, 82.47% reduction in transesophageal echocardiograms, 83.16% reduction in stress echo, 70.32% reduction in nuclear tests, 46.25% reduction in calcium score, 73.91% reduction in coronary computed tomography angiography, and 87.23% reduction in cardiac magnetic resonance imaging. TTE findings were overall similar between 2020 and 2019 (all *p* ≥ 0.05), except for a significantly higher right ventricular systolic pressure in 2020 (39.8 ± 14.2 vs. 34.6 ± 11.2 mmHg, *p* = 0.012). (4) Conclusions: Despite encountering an influx of critically ill patients, our hospital center experienced a reduction in the number of cardiac imaging studies, which likely represents a change in both patient mindset and physician management approach.

## 1. Introduction

The global novel coronavirus (COVID-19) pandemic caused a major outbreak in New York City (NYC), greatly affecting the delivery of healthcare. There has been increasing evidence that COVID-19 infection causes cardiac complications [1,2,3,4,5]. However, the pathophysiology and mechanisms of these complications of COVID-19 have not been fully elucidated. Acute myocardial injury, myocarditis, arrhythmias, and venous thromboembolism have been described and appear to be more prevalent in severe cases. Despite emerging evidence for troponin as a prognostic tool, COVID-19 patients have a wide range of myocardial injury due to multiple possible mechanisms and etiologies [6]. Echocardiography (echo) is the first-line imaging modality to assess cardiac morphology and function to help understanding clinical conditions and to guide to appropriate treatments. While echo is the fundamental diagnostic tool in our daily clinical practice, performing these studies increases the exposure of the sonographers and the rest of the echo laboratory staff to COVID-19. The American Society of Echocardiography (ASE) published guidelines to minimize exposure to COVID-19 and to reduce potential nosocomial transmission of the virus [7]. Recommendations included assessment of transthoracic echocardiogram (TTE) indication for appropriateness, utilization of personal protective equipment (PPE) during performance, and minimization of patients and staff in the echo laboratory [7]. Additionally, focused protocols may be implemented to answer specific clinical questions to decrease scan time. When considered together, these adjustments may have significant consequences on the operations and efficiency of an echo lab. We propose developing a quality assurance (QA) study to evaluate how the COVID-19 infection affected echo laboratory operations during the peak of the early 2020 wave of infections in NYC. Having a better understanding of the laboratory’s throughput has a direct impact on patient triage and addressing discharge barriers. During such a strained time for resources at a hospital, it is even more important to identify which studies require faster report times for improved clinical triage and disposition. The purpose of this study is to explore how the COVID-19 infection has affected echo lab operations, specifically regarding changes in echo study volume, study indications, turnaround time, and abnormal findings.

## 2. Materials and Methods

From the electronic medical record, we retrospectively identified all inpatient laboratory-performed TTE across three hospitals in the Montefiore Health System (Bronx, NY, USA) between 11 March 2020 to 5 May 2020 (when the number of COVID-19 cases in New York surged) and the same calendar period in 2019. The first consecutive 100 cases from 2019 were analyzed as a control group. The number of transesophageal echocardiograms (TEE), stress echo, nuclear tests, calcium score, coronary computed tomography angiography (CCTA), and cardiac magnetic resonance imaging (CMR) during these time periods were also identified. The TTEs were performed by echo sonographers or cardiology fellows for standard or limited follow-up evaluation with or without ultrasound enhancing agents or agitated saline injection. Information was obtained on patient age, sex, race, location of care (emergency department (ED), intensive care unit (ICU), or non-ICU floor), mechanical ventilation status, as well as the date and time when TTE was ordered, performed, and reported. From the TTE report, we collected information on the quality of TTE, left ventricular ejection fraction (LVEF), right ventricular (RV) systolic pressure (RVSP), and the presence of abnormal RV size, RV systolic function, pericardial effusion, valvular abnormalities including aortic stenosis (AS), aortic regurgitation (AR), mitral stenosis (MS), mitral regurgitation (MR), tricuspid stenosis (TS), tricuspid regurgitation (TR), and pulmonic regurgitation (PR). Wall motion abnormalities and diastolic function were not included in collection. The following criteria were used for abnormal thresholds: RV dilatation mild and above, RV hypokinesis mild and above, valvular stenosis mild and above, non-aortic valvular regurgitation (MR, TR, or PR) moderate or above, AR mild and above, EF < 51%, RVSP > 35 mmHg, and pericardial effusion small or above. If a TTE study had any of these abnormal findings, it was considered abnormal. The quality of the echo images was rated into four categories by the reading cardiologists: acoustic windows overall adequate, technically difficult study, technically difficult study with limited diagnostic information, or all cardiac structures poorly visualized. We considered the first two categories as a TTE study with good imaging quality, while the last two categories were considered as a TTE study with poor quality with limited diagnostic information. If a patient had a TTE study within 6 months, we compared the reports to determine whether there were any changes. For the patients who had TTE in 2020, we obtained the result of the COVID-19 polymerase chain reaction test (either Bioreference (Elmwood Park, NJ, USA) or Cepheid (Sunnyvale, CA, USA)) at the time when TTE was performed. For those whose COVID-19 results were pending at the time of TTE, the subsequent final COVID-19 test results were also obtained.

Patient characteristics were described across patients’ location and status of mechanical ventilation. Time-to-scan, time-to-report, TTE characteristics, and TTE findings were also analyzed in this study. Categorical variables were presented as count (proportion) and categorical data were compared using the Chi-square test or Fisher’s exact test. Parametric continuous variables were described as mean ± standard deviation (SD) and compared using Student’s *t*-test. Non-parametric continuous variables were described as median [interquartile range (IQR)] and compared using the Wilcoxon rank sum test. Comparisons were made as follows: (1) 2020 total vs. 2019, (2) 2020 COVID-19 positive vs. 2019, (3) 2020 COVID-19 negative vs. 2019, and (4) 2020 COVID-19 positive vs. COVID-19 negative. Patients with pending COVID-19 results at the time of TTE studies were included in the COVID-19 positive group because test performance followed the same protocol used for a confirmed positive test. The number of TTEs were assessed monthly during the study period and compared between 2019 and 2020 using a paired sample t-test. Finally, the number of other cardiovascular imaging modality studies were compared between 2020 and 2019. Statistical analysis was performed using SAS version 9.4 (SAS Institute Inc., Cary, NC, USA). A two-sided *p*-value of < 0.05 was considered statistically significant.

## 3. Results

Between 11 March 2020 and 5 May 2020, 1974 patients underwent TTE (Table 1). Of these, 924 patients (46.8%) tested positive for COVID-19. The distribution of race/ethnicity was similar between COVID-19 positive and negative groups. Higher percentages of COVID-19 positive patients were in the ICU (30.4 vs. 20.2%, *p* < 0.001) and mechanically ventilated (27.5 vs. 9.7%, *p* < 0.001), as compared to COVID-19 negative patients.

**Figure 1:**
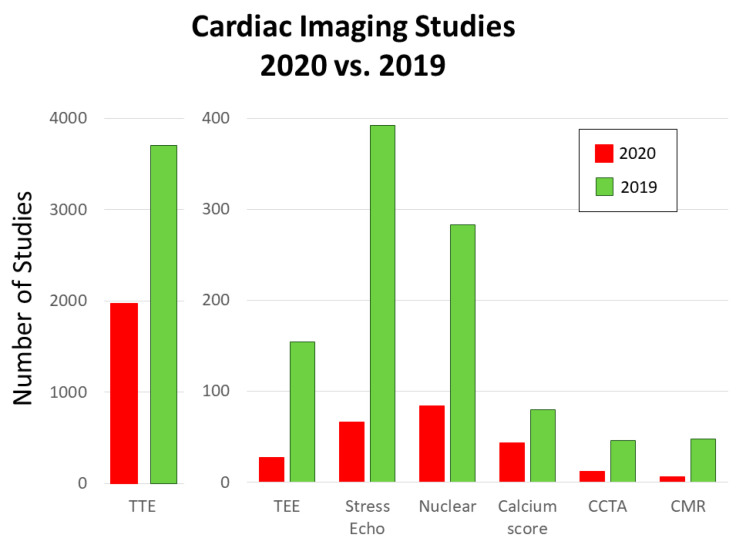
Number of cardiac imaging studies in 2020 and 2019 during the study period. The number of studies decreased during the COVID-19 pandemic compared with the corresponding period in 2019 in all the modalities including TTE, TEE, stress echo, nuclear tests, calcium score, CCTA, and CMR. CCTA, coronary computed tomography angiography; CMR, cardiovascular magnetic resonance; COVID, coronavirus; TEE, transesophageal echocardiography; TTE, transthoracic echocardiogram.

**Figure 2:**
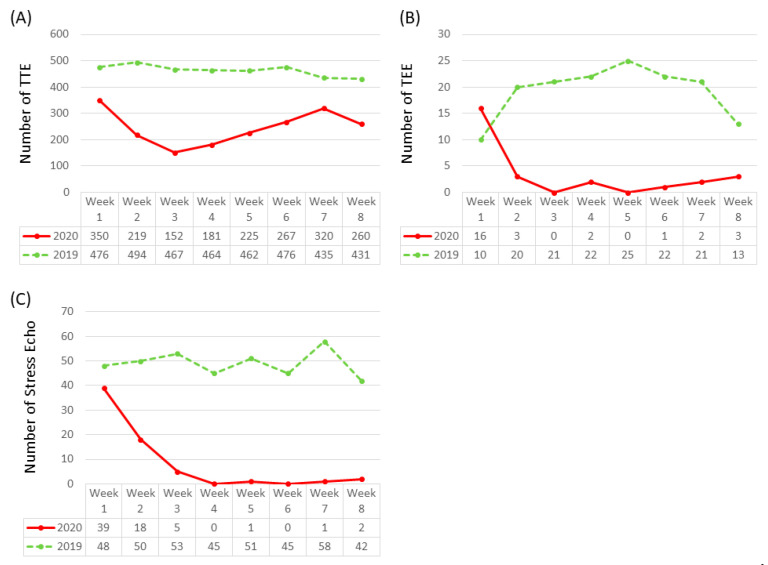
Weekly number of (**A**) TTE, (**B**) TEE, and (**C**) stress echocardiogram studies during the spring 2020 peak of the COVID-19 pandemic compared with the corresponding period in 2019. During the first three weeks of the COVID-19 pandemic, the number of studies declined in all three modalities. After the first three weeks, there were some fluctuations in numbers of TTE studies, whereas number of TEE and stress echocardiogram studies remained low. TEE, transesophageal echocardiography; TTE, transthoracic echocardiogram.

**Figure 3:**
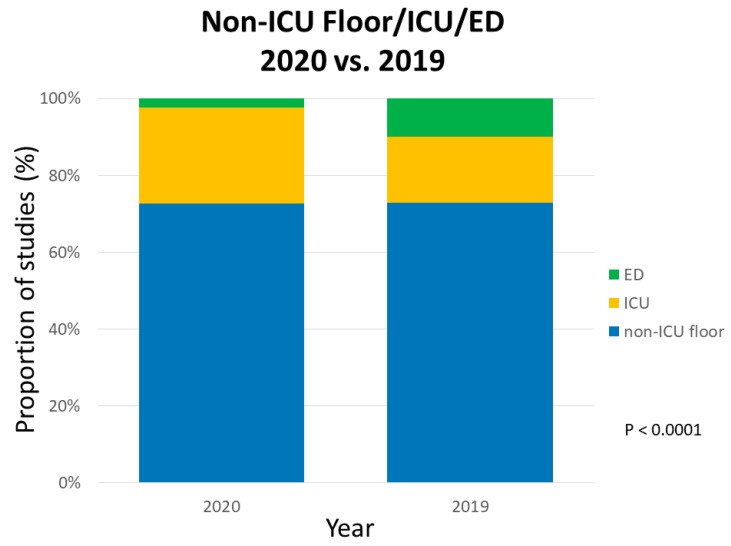
Proportion of TTE studies from non-ICU floor, ICU, and ED during the spring 2020 peak of the COVID-19 pandemic compared with the corresponding period in 2019. The proportion by location was significantly different between 2020 and 2019. ICU, intensive care unit; ED, emergency department.

The volume of studies in all cardiac imaging modalities including TTE, TEE, stress echo, nuclear tests, calcium score, CCTA, and CMR decreased during the COVID-19 pandemic compared with the corresponding period in 2019 (Figure 1). Our center experienced a 46.72% reduction in TTEs, 82.47% reduction in TEEs, 83.16% reduction in stress echo, 70.32% reduction in nuclear tests, 46.25% reduction in calcium score, 73.91% reduction in CCTA, and 87.23% reduction in CMR. In the first three weeks of the pandemic, the number of TTE, TEE, and stress echo decreased steadily to 32.5%, 0.0%, and 9.4% of their 2019 volumes, respectively (Figure 2). After the first three-week period, the 2020 TTE volume never exceeded 73.6% of the 2019 volume and only eight TEEs and four stress echos were performed. During the COVID-19 pandemic, there were 19 (29%) dobutamine stress echo and 47 (71%) exercise stress echo, whereas in 2019, there were 129 (33%) dobutamine stress echo and 263 (67%) exercise stress echo. The distribution of location of TTE studies (floor vs. ICU vs. ED) was statistically different between 2019 and 2020 (Figure 3).

Times of TTE order-to-perform, order-to-report, and perform-to-report are demonstrated in Table 2. Compared to 2019, these times in 2020 were significantly shorter in duration (all *p* < 0.01). When comparing time durations by location between 2019 and 2020, non-ICU floor TTEs had significantly shorter times in 2020, whereas the time durations for ICU and ED TTEs were similar in both years.

There was a trend towards a higher proportion of studies with poor TTE image quality during the 2020 pandemic (15.6%) compared to the sample in 2019 (11%), but this difference was not statistically significant (*p* = 0.21) (Table 3 and Table 4). Among 2020 studies, COVID-19 positive patients had a higher proportion of poor TTE image quality compared to COVID-19 negative patients (19.1 vs. 12.6%, *p* < 0.0001). The proportion of studies with poor TTE image quality in COVID-19 negative patients from 2020 was similar to the 2019 sample.

In terms of abnormal findings noted on TTE reports, there was a significantly higher proportion of studies with abnormal findings in 2020 COVID-19 negative patients compared to 2020 COVID-19 positive patients (48.4 vs. 42.2%, *p* = 0.0066). A similar proportion of studies with abnormal findings was found in COVID-19 negative patients in 2020 and the 2019 sample (48.4 vs. 51%, *p* = 0.61). In 2020, more COVID-19 negative patients had a prior TTE within 6 months compared to COVID-19 positive patients (27.3 vs. 16.8%, *p* < 0.0001).

Reported TTE findings were overall similar between 2020 and 2019 (all *p* ≥ 0.05), except for a significantly higher RVSP in 2020 studies (39.8 ± 14.2 vs. 34.6 ± 11.2 mmHg, *p* = 0.012) (Table 5). COVID-19 positive patients showed significantly higher RVSP compared to patients in 2019 (*p* = 0.0028), however the RVSP was similar between COVID-19 positive and COVID-19 negative patients within 2020 (*p* = 0.08). When comparing other TTE findings between COVID-19 positive and COVID-19 negative patients in 2020, COVID-19 negative patients had a significantly lower LVEF (*p* < 0.0001) and more hemodynamically significant MR, AR, AS, and TR (all *p* < 0.01, Table 5). There was no significant difference in proportion with reported RV dilation or RV hypokinesis between 2020 COVID-19 positive and COVID-19 negative patients.

## 4. Discussion

This study sought to explore how the COVID-19 pandemic has affected echo lab operations at a major academic center in NYC and to evaluate echo findings in COVID-19 patients. The volume of all cardiac imaging studies significantly decreased during the COVID-19 pandemic. The volume of echo steadily decreased over the first three weeks of the pandemic and remained low throughout the remaining five weeks examined. The performance and report times of TTEs during the pandemic were shorter. Finally, although overall results were similar between 2020 and 2019, COVID-19 negative patients showed significantly higher incidence of abnormal cardiac findings compared to COVID-19 positive patients.

The reduction in the number of cardiac imaging studies likely represents a change in both patient mindset and physician management approach. While the number of COVID-19 admissions surged, admissions for alternative diagnoses, particularly acute cardiac conditions, declined [8]. Reports have indicated that healthcare systems encountered a decline in acute coronary syndrome admissions [9,10,11,12]. Garcia et al. found that certain high volume cardiac catheterization laboratories in the US experienced a reduction in ST-elevation myocardial infarction activation of approximately 38% [9]. Congestive heart failure admissions declined as well [13,14]. It has commonly been hypothesized that patient fear of nosocomial COVID-19 transmission is a major factor in avoiding or delaying medical attention. Another possible reason for the reduction in cardiac imaging studies during the pandemic is increased judiciousness in physician ordering. In order to limit possible exposure to imaging technologists, teams may have relied more heavily on history, physical exam, and laboratory work to answer clinical questions. TEE requests were highly scrutinized because of the potential of aerosolization during the procedure. Lastly, the utilization of point-of-care ultrasound (POCUS) has become more commonplace on rounds for rapidly answering clinical questions [15,16]. Although it was not quantified, the implementation of POCUS likely contributed to the overall reduction in the number of cardiovascular imaging studies [17,18]. The ED and ICU teams, in particular, have the greatest access to POCUS. The ASE recommends the use of POCUS as a rapid screening tool, although TTE is often necessary to better characterize cardiac function or abnormalities. The economic impact of such a loss in cardiac imaging volume has yet to be determined, but is likely substantial [19,20,21].

The main contributor of increased echo lab operational efficiency (defined as improved order-to-perform, perform-to-report, and order-to-report times) during the pandemic was likely related to decreased TTE volume, which allowed staff to respond more quickly to the fewer requests. Implementation of a more active physician review of TTE studies has the potential to further reduce unnecessary TTE testing volume and thus, improve efficiency [22]. Another, probably minor, contributor was the removal of pre-reading by fellows-in-training (FITs), while they were redeployed to manage COVID-19 patients throughout the hospital system. While it is unknown what impact FITs have on report times, the educational value of such training far outweighs any possible “loss in efficiency”. Unfortunately, the pandemic may have a significant impact on FITs due to loss of educational experience [23]. Interestingly, there was no difference in times based on patient COVID-19 infection status, likely due to sonographers taking similar precautions regardless of patient COVID-19 status. Early in the pandemic, testing was unreliable and thus, PPE was recommended for every patient. As COVID-19 testing has improved and negative patients are more reliably identified, TTE waiting times for COVID-19 negative patients will likely decrease. The ED experienced the fastest performing and reporting times as these patients required rapid triaging and evaluation. Medicine and surgery floor patients encountered the slowest performing and reporting times. Such discrepancies in reporting times have important implications for optimizing patient flow and hospital throughput.

Interestingly, COVID-19 positive patients did not have a higher proportion of abnormal TTE findings compared to the COVID-19 negative cohort, possibly because those not suffering a COVID-19 infection may have deferred treatment until they were more severely ill. Furthermore, those requiring non-COVID-19 related inpatient care during the pandemic may have had more baseline cardiac illness. Compared to our COVID-19 positive patients, our COVID-19 negative patients had more TTEs within the previous six months, suggesting a history or suspected history of cardiac disease. Other studies have identified echocardiographic findings of COVID-19 patients, emphasizing the prevalence of RV dilation and dysfunction [24,25,26], which is thought to be due to a combination of hypoxemic pulmonary vasoconstriction, pulmonary embolus, decreased lung volume, increased positive end-expiratory pressure, hypercapnia, and a pro-inflammatory state. However, these studies only evaluated findings in a relatively small sample size of COVID-19 patients and did not compare to admitted COVID-19 negative patients during the same time frame. Our COVID-19 positive patients did not have a higher prevalence of RV dilation or dysfunction, but patients from 2020 had significantly higher RVSP than those from 2019. As more echo data related to COVID-19 infection becomes available, further research in this area may allow the scientific community to better identify and understand cardiac consequences of COVID-19.

This observational study of our cardiovascular imaging laboratory during the COVID-19 pandemic has some limitations. The retrospective nature of the analysis is limited by the data that were collected at the time. As an observational study, confounders affecting the results are possible. Although this analysis was performed at a high volume hospital system, it is a single center study based in an academic hospital, which may limit the generalizability of our findings. Lastly, COVID-19 testing reliability, which may have been worse early in the pandemic [27], could have resulted in misclassification and impacted our statistical analysis.

## 5. Conclusions

The spring 2020 COVID-19 pandemic peak in NYC had a substantial impact on hospital operations. Although encountering an influx of critically ill patients, our hospital center experienced a reduction in the volume of cardiac imaging studies. As the pandemic has become more controlled and cardiac imaging volume has returned to pre-pandemic levels, ongoing evaluation of operational flow and efficiency in the echo lab is important in optimizing patient care, especially with the possibility of further waves of COVID-19 infections.

## Figures and Tables

**Table 1:** Patient characteristics.

Variables	2019	2020						
	Total *n* = 100	Total *n* = 1974	*p*	COVID (+) or Suspected *n* = 924	*p*	COVID (−) *n* = 1050	*p*	p COVID (+) vs. (−)
Male	43 (43)	1108 (56.1)	0.010	523 (56.6)	0.0094	585 (55.7)	0.015	0.69
Age, yrs	64.7 ± 15.7	64.4 ± 15.0	0.84	63.8 ± 14.3	0.58	64.8 ± 15.5	0.92	0.13
Race/Ethnicity			0.33		0.26		0.31	0.0037
African American	35 (35)	711 (36.0)		322 (34.9)		389 (37.1)		
White	11 (11)	286 (14.5)		112 (12.1)		174 (16.6)		
Hispanic/Latino	36 (36)	648 (32.8)		323 (35.0)		325 (31.0)		
Asian	0 (0)	53 (2.7)		33 (3.6)		20 (1.9)		
Others	4 (4)	89 (4.5)		49 (5.3)		40 (3.8)		
Unknown	14 (14)	187 (9.5)		85 (9.2)		102 (9.7)		
Location			<0.0001		<0.0001		0.0023	<0.0001
medicine/surgery floor	73 (73)	1435 (72.7)		630 (68.2)		805 (76.7)		
ICU	17 (17)	493 (25.0)		281 (30.4)		212 (20.2)		
ED	10 (10)	46 (2.3)		13 (1.4)		33 (3.1)		
Mechanical ventilation	10 (10)	356 (18.0)	0.040	254 (27.5)	0.0001	102 (9.7)	0.93	<0.0001

**Table 2:** Time to TTE perform and report.

Variables	2019		2020									
	Total		Total			COVID (+) or Suspected			COVID (−)			*P* COVID (+) vs. (−)
	*n*	Median [IQR]	*n*	Median [IQR]	*p*	*n*	Median [IQR]	*p*	*n*	Median [IQR]	*p*	
Order-to-perform time, h	100	15.3 [4.7, 31.4]	1972	10.8 [2.7, 20.2]	0.0032	923	11.2 [2.8, 20.9]	0.0065	1049	10.4 [2.6, 19.6]	0.0026	0.39
medicine/surgery floor	73	17.2 [5.5, 36.7]	1434	12.4 [3.1, 21.3]	0.0026	629	13.1 [3.2, 21.9]	0.0056	805	11.6 [3.1, 20.7]	0.0022	0.43
ICU	17	12.5 [9.3, 19.3]	492	9.1 [2.0, 18.9]	0.082	281	8.2 [2.2, 19.4]	0.10	211	9.2 [1.8, 18.4]	0.069	0.54
ED	10	3.9 [1.6, 10.2]	46	4.1 [1.1, 10.4]	0.90	13	4.3 [1.1, 15.8]	0.93	33	3.8 [1.5, 7.3]	0.82	0.82
Order-to-report time, h	100	19.0 [6.3, 32.9]	1974	14.0 [4.8, 22.9]	0.0007	924	14.4 [4.9, 23.5]	0.0015	1050	14.0 [4.7, 22.3]	0.0006	0.64
medicine/surgery floor	73	21.1 [6.8, 40.7]	1435	14.8 [5.2, 23.8]	0.0004	630	15.5 [5.2, 24.7]	0.0009	805	14.5 [5.2, 23.4]	0.0004	0.75
ICU	17	17.6 [13.8, 23.6]	493	12.1 [3.9, 21.8]	0.062	281	11.6 [4.3, 22.1]	0.080	212	12.8 [3.9, 21.3]	0.053	0.68
ED	10	5.7 [2.4, 12.2]	46	7.9 [3.9, 14.0]	0.68	13	8.8 [4.2, 18.3]	0.48	33	6.7 [3.4, 12.0]	0.83	0.83
Perform-to-report time, h	100	2.1 [1.4, 3.1]	1972	1.7 [1.2, 2.4]	0.0010	923	1.6 [1.2, 2.2]	0.0002	1049	1.7 [1.2, 2.5]	0.0055	0.014
medicine/surgery floor	73	2.1 [1.3, 3.0]	1434	1.7 [1.2, 2.3]	0.014	629	1.6 [1.2, 2.2]	0.0033	805	1.7 [1.2, 2.5]	0.046	0.011
ICU	17	3.0 [2.2, 3.7]	492	1.6 [1.1, 2.4]	0.0031	281	1.6 [1.1, 2.3]	0.0023	211	1.6 [1.1, 2.5]	0.0067	0.42
ED	10	1.9 [1.1, 2.0]	46	2.1 [1.3, 2.8]	0.29	13	2.3 [2.1, 3.3]	0.028	33	1.7 [1.2, 2.4]	0.70	0.70

**Table 3:** TTE characteristics.

Variables	2019	2020						
	Total *n* = 100	Total *n* = 1974		COVID (+) or Suspected *n* = 924		COVID (−) *n* = 1050		*P* COVID (+) vs. (−)
	*n* (%)	*n* (%)	*p*	*n* (%)	*p*	*n* (%)	*p*	
Image quality			0.21		0.048		0.65	<0.0001
Excellent or good	89 (89)	1660 (84.4)		748 (81.0)		912 (87.4)		
Poor	11 (11)	307 (15.6)		176 (19.1)		131 (12.6)		
Abnormal results	51 (51)	898 (45.5)	0.28	390 (42.2)	0.092	508 (48.4)	0.62	0.0066
Echo within 6 months	23 (23)	442 (22.4)	0.89	155 (16.8)	0.12	287 (27.3)	0.35	<0.0001

**Table 4:** Distribution of abnormal results in prior TTE.

Variables	2019	2020						
	Total *n* = 23	Total *n* = 441		COVID (+) *n* = 154		COVID (−) *n* = 287		*p* COVID (+) vs. (−)
	*n* (%)	*n (%)*	*p*	*n* (%)	*p*	*n* (%)	*p*	
Abnormal results in prior TTE	12 (52.2)	308 (69.8)	0.074	94 (61.0)	0.42	214 (74.6))	0.020	0.0045

**Table 5:** TTE results.

Variables	2019		2020									
	Total		Total			COVID (+)			COVID (−)			*p* COVID (+) vs. (−)
	*n*		*n*		*p*	*n*		*p*	*n*		*p*	
LVEF, %	94	60 [55, 65]	1777	60 [50, 65]	0.12	728	60 [55, 65]	0.58	1049	60 [45, 65]	0.030	0.0001
Abnormal valve findings	98	20 (20.4)	1855	369 (19.9)	0.90	768	108 (14.1)	0.096	1087	261 (24.0)	0.42	<0.0001
Mitral regurgitation		7 (7.1)		127 (6.9)	0.91		27 (3.5)	0.08		100 (9.2)	0.50	<0.0001
Aortic insufficiency		5 (5.1)		103 (5.6)	0.85		23 (3.0)	0.27		80 (7.4)	0.41	<0.0001
Aortic stenosis		6 (6.1)		49 (2.6)	0.042		17 (2.2)	0.023		32 (2.9)	0.087	0.33
Tricuspid regurgitation		5 (5.1)		151 (8.1)	0.28		44 (5.7)	0.80		107 (9.8)	0.12	0.0014
Pulmonary regurgitation		0 (0.0)		15 (0.81)	1.00		3 (0.4)	1.00		12 (1.1)	0.61	0.11
Possible endocarditis	98	1 (1.0)	1856	10 (0.54)	0.43	768	5 (0.7)	0.51	1088	5 (0.46)	0.40	0.75
RV dilatation	98	13 (13.3)	1714	259 (15.1)	0.62	690	108 (15.7)	0.54	1024	151 (14.8)	0.69	0.61
RV hypokinesis	97	97 (17.5)	1564	267 (17.1)	0.94	644	107 (16.6)	0.86	920	160 (17.4)	0.99	0.69
RVSP	39	32 [28. 40]	300	38 [30, 47]	0.016	101	40 [31, 50]	0.0047	199	38 [29, 45]	0.050	0.10
Pericardial effusion	98	3 (3.1)	1873	72 (3.8)	0.69	776	31 (4.0)	0.65	1097	41 (3.7)	0.73	0.78
